# Maternal Infections, Antibiotics, Steroid Use, and Diabetes Mellitus Increase Risk of Early-Onset Sepsis in Preterm Neonates: A Nationwide Population-Based Study

**DOI:** 10.3390/pathogens14010089

**Published:** 2025-01-17

**Authors:** Hao-Yuan Lee, Yu-Lung Hsu, Wen-Yuan Lee, Kuang-Hua Huang, Ming-Luen Tsai, Chyi-Liang Chen, Yu-Chia Chang, Hung-Chih Lin

**Affiliations:** 1 Department of Nursing, Jen-Teh Junior College of Medicine, Nursing and Management, No. 79-9, Sha-Luen-Hu, Xi-Zhou Li, Hou-Loung Town, Miaoli 35664, Taiwan; 2 Department of Pediatrics, Wei Gong Memorial Hospital, Miaoli 35159, Taiwan; 3 School of Medicine, College of Medicine, Fu Jen Catholic University, New Taipei 242062, Taiwan; 4 Division of Pediatric Infectious Diseases, China Medical University Children’s Hospital, China Medical University, Taichung 40447, Taiwan; codan5230@gmail.com; 5 Department of Neurosurgery, Wei Gong Memorial Hospital, Miaoli 35159, Taiwan; 005122@tool.caaumed.org.tw; 6 Department of Neurosurgery, China Medical University Hospital, China Medical University, Taichung 40447, Taiwan; 7 Department of Health Services Administration, China Medical University, Taichung 40447, Taiwan; khhuang@mail.cmu.edu.tw; 8 Division of Neonatology, China Medical University Children’s Hospital, China Medical University, Taichung 40447, Taiwan; williamtsai1012@gmail.com; 9 Molecular Infectious Disease Research Center, Chang Gung Memorial Hospital at Linkou, Taoyuan 333423, Taiwan; dinoschen@cgmh.org.tw; 10 Department of Long Term Care, College of Health and Nursing, National Quemoy University, No. 1, University Road, Jinning Township, Kinmen County 892009, Taiwan; 11 Department of Healthcare Administration, College of Medical and Health Science, Asia University, Taichung 41354, Taiwan; 12 Department of Pediatrics, Asia University Hospital, Asia University, Taichung 41300, Taiwan

**Keywords:** early-onset sepsis, risk factors, maternal infections, antibiotic and steroid use, diabetes mellitus

## Abstract

The global evolution of pathogens causing early-onset sepsis (EOS), a critical condition in preterm infants, necessitates a re-evaluation of risk factors to develop updated prevention and treatment strategies. This nationwide case–control study in Taiwan analyzed data from the National Health Insurance Research Database, Birth Reporting Database, and Maternal and Child Health Database from 2010 to 2019. The study included 176,681 mother–child pairs with preterm births. We identified 2942 clinical EOS cases from 5535 diagnosed sepsis cases, excluding unlikely cases. A control group of 14,710 preterm neonates without EOS was selected at a 1:5 ratio. Clinical EOS increased since 2017. Adjusted logistic regression identified significant EOS risk factors in preterm infants, including maternal fever, chorioamnionitis, maternal diabetes mellitus, maternal antibiotic usage, very preterm birth, birth weight (all with *p* < 0.001), maternal pneumonia (*p* = 0.002), and maternal CS (*p* = 0.004). Effective treatment of maternal conditions like diabetes, fever, and infections is essential to prevent EOS in preterm infants. Key measures include reducing unnecessary antibiotics or steroids, minimizing unnecessary cesarean sections, avoiding premature or prolonged rupture of membranes (PPROM), and increasing gestational age and neonatal birth weight. High-risk preterm neonates should be closely monitored for EOS and considered for antibiotics when warranted.

## 1. Introduction

The observed rise in early-onset sepsis (EOS) caused by *E. coli* infections in preterm infants in the United States (US) between 2015 and 2017, and by *Listeria monocytogenes* infections in Taiwan from 2012 to 2018, highlights the urgent need for further research to re-evaluate risk factors and develop innovative prevention and treatment strategies for EOS [1,2]. Identifying risk factors for sepsis, a severe condition characterized by life-threatening organ dysfunction due to a dysregulated host response to pathogens, provides opportunities for early detection and intervention [3,4]. Prompt initiation of appropriate antimicrobial therapy in high-risk patients is paramount for improving sepsis outcomes [5].

EOS remains a serious and often fatal illness among infants born preterm, particularly those of the lowest gestational age [6,7,8]. EOS among very preterm infants has a contemporary incidence of approximately 13–18 cases per 1000 preterm births at less than 29 weeks gestation, with an associated all-cause mortality rate as high as 35%, making it one of the leading causes of neonatal morbidity and mortality [9,10].

Most preterm infants with very low birth weight (VLBW) are currently treated empirically with antibiotics for the risk of EOS, often for prolonged periods, even in the absence of a culture-confirmed infection [11,12]. Retrospective studies have revealed that antibiotic exposure after birth is associated with multiple subsequent poor outcomes among preterm infants, making the risk/benefit balance of these antibiotic treatments uncertain [8].

Preterm EOS is defined as a blood or cerebrospinal fluid (CSF) culture obtained within 72 h after birth that grows a pathogenic bacterial species [8], as it can lead to severe complications such as septic shock, organ dysfunction, and death if not promptly treated [5,6,7,8]. Neonatal EOS typically refers to an invasive bacterial infection detected within the first 72 h of life in infants [9,13].

In Taiwan, we collect all clinical sepsis cases diagnosed by specialized physicians and exclude unlikely cases, including those lacking blood culture, antibiotic treatment, or admission for less than 10 days. This decision aligns with the American Academy of Pediatrics (AAP) guidelines, which advocate for a minimum of 10 days of antibiotic treatment for neonatal culture-confirmed sepsis [14].

The AAP guidelines outlined low-risk criteria for EOS to identify newborns at risk of sepsis and prevent unnecessary antibiotic therapy initiation [12]. However, the rise in certain EOS pathogens highlights the need for further research to reassess risk factors and develop innovative prevention and treatment strategies. Numerous studies have explored maternal and neonatal risk factors linked to EOS. However, several risk factors remain contentious across studies, and no nationwide study has provided comprehensive clarification due to limited case volumes. In addition, our clinical observations revealed that maternal infections, antibiotic or steroid usage, underlying diseases, or cesarean section (CS) seem to increase the risk of EOS in term newborns. Therefore, we designed this nationwide case–control study to determine these risk factors in full-term EOS. Term newborns were deliberately excluded from this analysis to prevent confounding effects.

## 2. Materials and Methods

### 2.1. Data Sources

In this nationwide case–control study, data from the National Health Insurance Research Database (NHIRD), Birth Reporting Database (BCD), and Maternal and Child Health Database (MCHD) were utilized to identify all pregnant individuals and their offspring in Taiwan spanning from 2010 to 2019. These databases were made available under license by the Health and Welfare Data Science Center, Ministry of Health and Welfare Taiwan (HWDC, MOHW). The National Health Insurance Program encompasses approximately 23 million individuals, representing nearly 99.9% of the Taiwanese population. The NHIRD contains validated diagnostic records, medication usage, and healthcare facility utilization. The BCD includes perinatal information such as gestational age and birth weight of newborns in Taiwan, while the MCHD contains de-identified identity numbers of newborns and their parents. De-identified medical claims records and prescription data from the NHIRD, BCD, and MCHD were utilized in this study. Specifically, the NHIRD was linked with the MCHD and the BCD to extract pertinent medical information regarding maternal and neonatal risk factors associated with early-onset sepsis.

### 2.2. Ethical Statements

This study received approval from the Institutional Review Board of the China Medical University Hospital, Taiwan (Institutional Review Board No. CMUH113-REC1-085). Informed consent was not necessary, as the databases utilized in this study solely contained de-identified data.

### 2.3. Study Subjects

**Figure 1:**
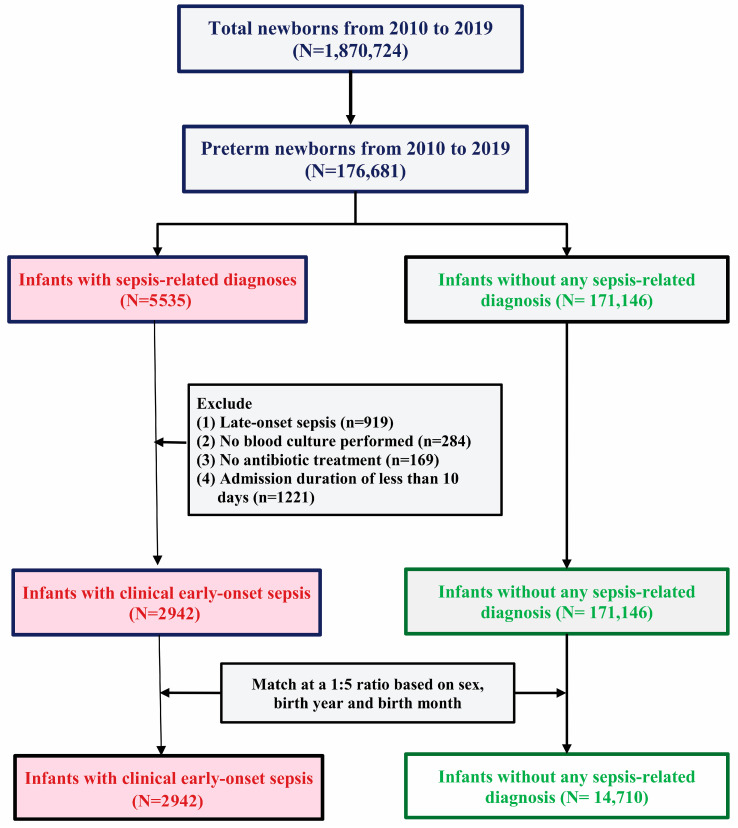
A flowchart outlining the selection process for both the clinically early-onset sepsis group and the non-sepsis control group, ensuring a 1:5 ratio match between the two groups in this study.

Between 2010 and 2019, we identified pairs of pregnant individuals and their term offspring, collecting all sepsis cases diagnosed by specialized physicians. To identify inpatient diagnoses, we used ICD-9-CM (International Classification of Diseases, Ninth Revision, Clinical Modification) codes prior to 2015 and ICD-10-CM codes thereafter (Table 1). As blood culture results were unavailable from the NHIRD, BCD, and MCHD databases, sepsis cases were identified by ICD-9-CM and ICD-10-CM codes. The epidemiology of sepsis and its associated mortality was calculated in newborns from birth to 90 days old. Clinical sepsis cases were defined by excluding all unlikely cases, such as those lacking blood culture, without antibiotic treatment, or admitted for fewer than 10 days, as clinical sepsis caused by bacteria typically requires antibiotic treatment and admission for at least 10 days. This approach follows American Academy of Pediatrics (AAP) guidelines, which generally recommend 10 days of treatment for neonatal bacteremia without a clear focus of infection [14]. Late-onset sepsis (≥72 h after birth) was excluded. Controls were matched by birth year and month at a 1:5 ratio from all live births, excluding individuals ever diagnosed with sepsis or admitted to the neonatal intensive care unit (NICU) or sick baby room to exclude neonates with sepsis (Figure 1, Table 2). Primary outcomes centered on EOS risk factors, analyzed by comparing data between the EOS and control groups.

### 2.4. Risk Factors

Maternal fever, maternal chorioamnionitis, maternal genitourinary tract infections, maternal group B streptococcus (GBS), and maternal pneumonia were defined as diagnoses made by ICD-9-CM or ICD-10-CM codes (refer to Table 1) at least once within 14 days prior to labor. Maternal systemic lupus erythematosus (SLE) and diabetes mellitus (DM) were defined as diagnoses made by ICD-9-CM or ICD-10-CM codes (refer to Table 1) at least once within 100 days prior to delivery. Maternal antibiotic or steroid usage was defined as receiving at least one type of antibiotic or steroid within 14 days prior to labor. Antibiotics were identified based on their ATC (Anatomical Therapeutic Chemical) codes, specifically J01 for antibacterial agents. Similarly, steroids were identified under H02AB01 for systemic rinderon and H02AB02 for dexamethasone.

### 2.5. Statistical Analysis

Statistical analysis was conducted using SAS software version 9.4 (SAS Institute, Cary, NC, USA). Statistical significance was determined at a *p* value < 0.05. Descriptive statistics were initially employed to illustrate the distributions and incident rates (IR) of various maternal and neonatal risk factors. Additionally, risk factors were examined using conditional logistic regression analysis with a stepwise selection method, adjusting for all control variables. The adjusted odds ratios (aORs) and corresponding 95% confidence intervals (CIs) were calculated accordingly.

## 3. Results

### 3.1. Epidemiology of Diagnosed Sepsis Among First 90 Days After Birth

Out of 1,870,724 neonates born between 2010 and 2019, a total of 18,871 newborns (1.01%) were diagnosed with sepsis by physicians within 90 days after birth. Among the 176,681 preterm newborns (9.45% of all neonates) born during this period, 5535 newborns (3.13%) experienced sepsis. The majority of sepsis cases (83.40%) occurred within the first three days after birth. Among the 4058 deceased term infants within 90 days after birth, 146 deaths (3.60%) were attributed to sepsis. The mortality rate among young preterm infants with sepsis (2.64%) was higher than that among those without sepsis (2.29%).

### 3.2. Selecting Clinical Early-Onset Sepsis Cases for Case–Control Study

A total of 2942 clinical cases of EOS were identified from a pool of 5535 diagnosed sepsis cases. Exclusions were made for cases lacking blood culture, those not receiving antibiotic treatment, and those with hospital stays of fewer than 10 days (Figure 1).

### 3.3. Baseline Characteristics of the Study Participants

**Figure 2:**
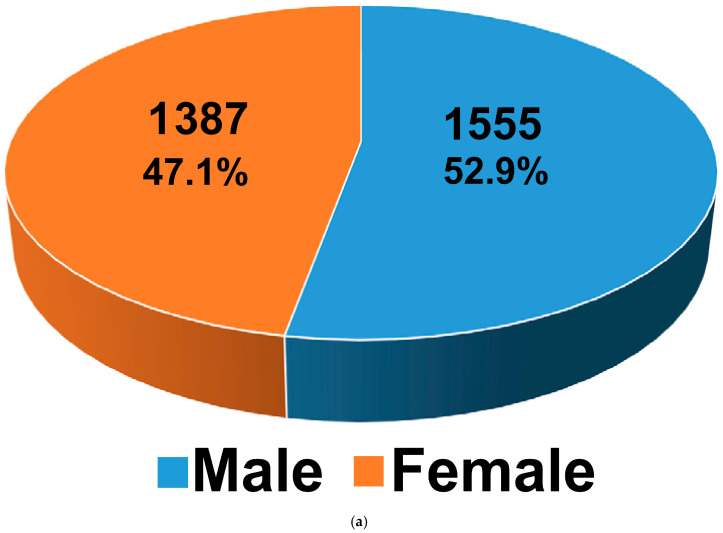

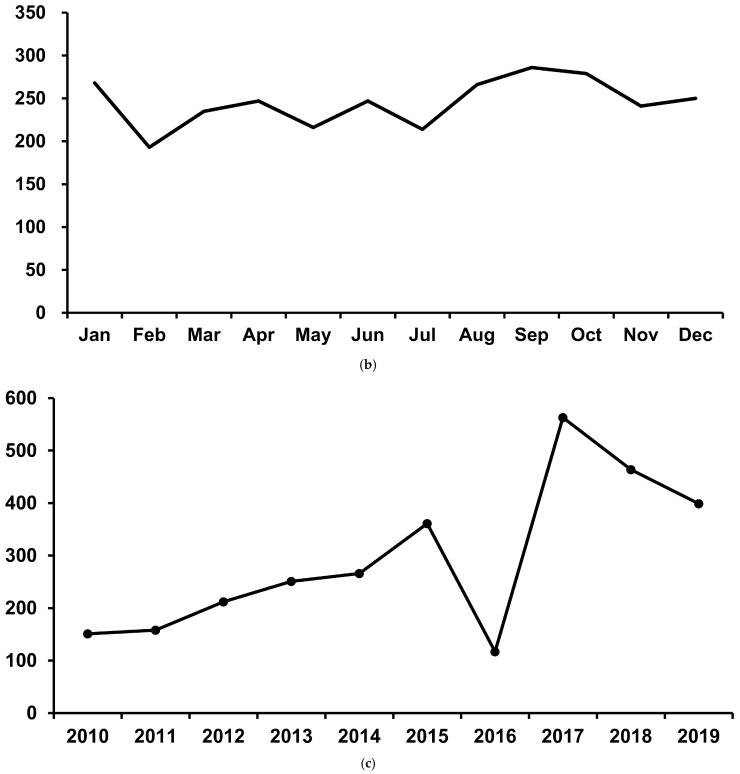
Distribution of clinical sepsis case numbers by gender (**a**), birth year (**b**), and birth month (**c**).

In a case–control study analyzing the risk factors for preterm infants acquiring EOS (n = 2942), a matching ratio of 1:5 was employed for the sepsis group and the non-sepsis group. No significant differences were observed between the data of the sepsis group and the non-sepsis group in terms of sex, birth year, or birth month (all *p* = 1). The distribution of clinical sepsis cases by gender, birth year, and birth month is illustrated in Figure 2.

### 3.4. Risk Factors of Preterm Infants Acquiring Sepsis

Preterm infants with EOS showed correlations with maternal fever, chorioamnionitis, genitourinary tract infections, pneumonia, premature/prolonged rupture of membranes, cesarean section (CS) delivery, maternal diabetes mellitus, maternal antibiotic usage, maternal steroid (rinderon or dexamethasone) usage, gestational age, birth weight (all with *p* < 0.001), and maternal DM (*p* = 0.008) based on Chi-square analysis. (Table 3).

Adjusted stepwise logistic regression revealed that preterm infants with EOS were associated with maternal fever, chorioamnionitis, maternal diabetes mellitus, maternal antibiotic usage, very preterm, birth weight (all with *p* < 0.001), maternal pneumonia (*p* = 0.002), and maternal CS (*p* = 0.004) (Table 4).

## 4. Discussion

Our study found that EOS rates did not decrease from 2010 to 2019 (Table 2) and even increased since 2017 [1]. This may be due to a rise in non-GBS infections, underscoring the urgency for further research to identify risk factors and develop new prevention strategies [1,2].

Based on this nationwide study, which includes the largest number of EOS cases in preterm infants globally, ten significant risk factors were identified: maternal fever, specific maternal infections (chorioamnionitis and pneumonia), maternal antibiotic or steroid use, maternal diabetes mellitus, cesarean section, premature or prolonged rupture of membranes, gestational age, and low birth weight. Further details on these findings are discussed below.

Maternal infections during the peripartum period are among the top three causes of maternal mortality globally. These infections also contribute to about half of preterm births and increase the risk of EOS [8,15,16,17,18]. While maternal fever has been linked to NS, its role in premature EOS is less clear. Only 26% of infants born at 22–36 weeks of gestation with culture-proven early-onset sepsis had mothers with a fever ≥38.0 °C [8,19,20]. Our study is the first to show that maternal fever is significantly associated with the highest adjusted odds ratio for the risk of EOS in preterm neonates.

Chorioamnionitis is strongly associated with EOS in preterm infants and had the third highest odds ratio for EOS risk in our study [8,21]. Genitourinary tract infections (UTIs) affect up to 41% of women of reproductive age globally and are significantly associated with EOS [17,18]. However, our study did not find a correlation between EOS and UTIs in preterm neonates, possibly due to effective maternal antibiotic treatment for these infections in Taiwan, as indicated by the overprescription of antibiotics in our results.

Pneumonia is the leading cause of fatal non-obstetrical infections during pregnancy, with incidences reported at 0.5–1 per 1000 pregnancies for all neonates in the USA and 3.69 per 1000 pregnancies for preterm neonates in our study [22]. Fetal outcomes associated with pneumonia during pregnancy include preterm birth, small for gestational age, fetal distress, intrauterine infection, EOS, and potential fetal mortality [23]. Previous studies have shown higher rates of early-onset neonatal infections in newborns of mothers with bacterial infections or colonization compared to those without such conditions. Our research is the first globally to identify a correlation between maternal pneumonia and an increased risk of preterm EOS, presenting the second highest odds ratio.

Preterm prelabor rupture of membranes (PPROM) complicates up to 3–4% of pregnancies and is associated with 30–50% of preterm births [24,25,26]. PPROM can lead to the proliferation of bacteria in the birth canal, resulting in their migration to the amniotic sac and potential infection [27]. Approximately 30–80% of preterm deliveries after PPROM are complicated by chorioamnionitis [28]. These factors were identified as correlates of EOS in our study, consistent with previous research [29]. Following the diagnosis of PPROM, an antibiotic (preferably erythromycin) should be administered for 10 days or until the onset of established labor, whichever occurs sooner [30].

To our knowledge, this study is the first to find that maternal antibiotic use increases the risk of preterm EOS. Antibiotics are commonly administered shortly after birth to nearly all preterm infants with very low birth weight (VLBW) (birth weight < 1500 g) as a precaution against EOS. This practice disrupts the maternal vaginal microbiota, which subsequently affects the establishment of the newborn’s gut microbiota. Consequently, there is a significant reduction in Lactobacillus species, potentially influencing the occurrence of EOS [31].

This study examined maternal bacterial infections, including chorioamnionitis, genitourinary tract infections, GBS colonization, and maternal pneumonia within 14 days before labor. These infections were present in 424 (14.41%) of the sepsis group and 816 (5.55%) of the non-sepsis group. Antibiotics were administered to 1426 (48.47%) mothers in the sepsis group and 4655 (31.65%) in the control group. Maternal antibiotics are indicated to be prescribed for bacterial infections and PPROM, with about 50% of PPROM cases overlapping with chorioamnionitis [28]. Consequently, many mothers received antibiotics without confirmed indications. Prophylactic antibiotics, without bacterial diagnosis or PPROM, were given to 438 (14.87%) mothers in the sepsis group and 2347 (15.95%) in the control group over the past decade in Taiwan. Reducing unnecessary antibiotic use by about 15% could potentially lower the risk of EOS.

A prior nationwide cohort study found that infants, both preterm and term, exposed to a single course of antenatal corticosteroids had a significantly higher likelihood of experiencing serious infections within the first 12 months of life [32]. However, the correlation between maternal steroid usage and EOS specifically among preterm neonates was previously unknown. Our study is the first in the world to show a significant association between these factors.

In this investigation, maternal underlying conditions were also assessed. Gestational diabetes mellitus (DM) is recognized for its contribution to adverse maternal, perinatal, and neonatal outcomes [33]. We noted an elevated incidence of EOS in preterm neonates linked to maternal DM, a novel finding in the existing literature. Conversely, mothers diagnosed with systemic lupus erythematosus (SLE), who face a heightened risk of peripartum infections and exposure to antibiotics, did not demonstrate a correlation with increased EOS in our study [34].

The relationship between CS and EOS has remained unclear, as previous studies lacked sufficient cases to establish an association [29]. The AAP guidance for EOS management in infants under 35 weeks gestation emphasizes delivery characteristics to identify preterm infants at lower EOS risk. This lower-risk category includes cesarean births for non-infectious indications, absence of labor, and no rupture of membranes (ROM) at delivery [9].

A retrospective cohort study conducted at four perinatal centers in the United States from 2017 to 2021 examined infants born before 35 weeks’ gestation. Out of the cohort, only 21 infants (0.7%) had EOS. Notably, no EOS cases were found among infants born by CS without ROM (with or without labor) or via CS with ROM for less than 18 h without labor. The authors suggested that larger preterm infant cohorts are needed to validate delivery phenotype-specific EOS risk [9].

However, a nationwide cohort study conducted in Korea revealed that neonatal sepsis rates were significantly higher in CS deliveries compared to vaginal deliveries (VD) for infants born at 27–28 gestational weeks (GW) (25% vs. 21%) and 29–34 GW (12% vs. 8%) between 2013 and 2017. No significant difference was observed in the 23–24 GW and 25–26 GW groups. Multivariate analyses showed that the adjusted odds ratios (ORs) for both sepsis and EOS were not significantly reduced with CS compared to VD [35].

Three factors may explain the differing results among these countries. First, intrauterine infections like sepsis can progress to EOS post-birth, inducing fetal inflammatory response syndrome (FIRS) [36]. This may lead to fetal distress, a common indication for emergency CS, which elevates EOS risk (OR = 3.00, *p* < 0.01) [37]. Additionally, elective CS is only 0.15 times less risky than emergency CS (*p* < 0.001) [38]. In the US, the elective CS rate (5.50%) is 9.82 times higher than Taiwan’s (0.56%, calculated as 12 out of 2125), potentially lowering EOS risk [39,40]. Second, advanced medical technology and stringent infection control measures during CS in the US may decrease EOS risk compared to vaginal delivery. Third, CS is considered a potential risk factor for EOS as it can disrupt the neonatal normal flora. Infants born via CS typically have lower levels of beneficial bacteria like *Bifidobacterium* and *Bacteroides* spp., which are vital for a healthy microbiome [19]. A balanced normal flora strengthens the immune system, and CS-induced alterations may increase EOS risk, as supported by our study. CS has also been linked to higher infection-related hospitalizations in offspring up to age 5 compared to vaginal birth [41,42]. Direct inoculation of the maternal microbiome during vaginal delivery may crucially impact early protective mucosal immunity in the gastrointestinal and respiratory tracts, affecting EOS risk [43].

Our study is the first to highlight that higher birth body weight can act as a protective factor against neonatal sepsis, especially among preterm infants. This finding holds particular significance as previous studies did not distinctly exclude term neonates [38]. Because gestational age is the strongest predictor of EOS and approximately two-thirds of preterm births are associated with preterm labor, PROM, or chorioamnionitis, gestational age is significantly influenced by these factors as well as birth weight in the adjusted logistic regression. In this study, extremely preterm infants (gestational age < 28 weeks) initially showed a significant correlation with EOS compared to those >32 weeks. However, this association became non-significant after adjusting for these factors in logistic regression due to the strong influence of other risk factors.

This study’s strengths lie in its comprehensive design, utilizing a nationwide database covering 176,681 mother–child pairs over a decade for detailed EOS risk factor analysis. Leveraging Taiwan’s universal single-payer healthcare system enabled thorough identification of diagnoses, prescriptions, and procedures, minimizing biases. Additionally, the substantial sample size provided robust statistical power for both cohort and sibling-matched analyses, enhancing the study’s reliability.

While our study provides valuable insights, it is subject to certain limitations. Firstly, the data were derived from a single country, necessitating validation across diverse populations to ensure generalizability. Secondly, the absence of blood culture results and comprehensive clinical data, such as symptoms and signs, in the National Health Insurance Research Database restricted the robustness of the study design. Reliance on discharge diagnoses and International Classification of Diseases (ICD) codes introduced potential biases due to coding errors and misclassification. Thirdly, patient selection may have influenced the outcomes, as we excluded 127 neonates who died from sepsis within the first 10 days after birth, accounting for 0.0719% of all preterm neonates, due to our exclusion criteria. This exclusion may have introduced a slight bias, although it minimally affected the overall study results. Lastly, additional factors such as regional healthcare disparities, variations in antibiotic practices, and differences in sepsis diagnosis criteria were not accounted for, potentially affecting the findings. Future studies should address these methodological and data limitations to provide more comprehensive insights.

## 5. Conclusions

Our study identified key maternal and neonatal factors associated with an increased risk of early-onset sepsis (EOS) in preterm infants. These include maternal conditions such as diabetes, fever, infections (e.g., chorioamnionitis and pneumonia), and the use of antibiotics or steroids. Neonatal factors such as very preterm birth, low birth weight, and preterm premature rupture of membranes (PPROM) were also significant contributors. Strategies to mitigate EOS risk should prioritize optimal maternal health management, reduction of unnecessary medical interventions, and the promotion of higher gestational age and birth weight. Preterm neonates with these risk factors require vigilant monitoring and timely intervention to reduce the incidence and severity of EOS.

## Figures and Tables

**Table 1:** Diagnoses of Sepsis and Risk Factors.

Sepsis or Risk Factors	ICD-9-CM Codes (Before 2015)	ICD-10-CM Codes (After 2015)
Sepsis	038.xx, 785.52, 998.02, 995.91, 995.92, 003.1, 036.2, 098.89, 771.81	A40.x, A41.xx, R65.20, R65.21, T81.12XA, T81.12XD, T81.12XS, A02.1, A39.4, A54.86, P36
Maternal risk factors		
Maternal fever	780.6, 659.20, 659.21, 659.23	R50. 9, O75.2
Chorioamnionitis	658.4x, 762.7	O41.101x, O41.102x, O41.103x, O41.1090, O41.121x, O41.122x, O41.123x, O41.1290, P02.7
Genitourinary tract infections	646.6x, 599.0	O23.xx, N39.0
Pneumonia	480.x–486	J12.xx-J18.x
Premature/prolonged rupture of membranes (PPROM)	658.1x, 658.2x	O42.xxx
Delivery by Cesarean section	669.70, 669.71, V30.01, V31.01, V32.01, V33.01, V34.01, V35.01, V36.01, V37.01, V39.01	O82, Z38.01, Z38.31, Z38.62, Z38.64, Z38.66, Z38.69, DRG 765, DRG 766
Maternal systemic lupus erythematosus (SLE)	710.0	M32.xx
Maternal diabetes mellitus	250.xx, 648.0x	E08.xxx-E13.xxx, O24.xxx

**Table 2:** Baseline Characteristics of Participants by Matching 1: 5 Ratio in this Case–control Study.

Variables	Clinical Sepsis Group (N = 2942)	Non-Clinical Sepsis Group (N = 14,710)	*p* Value
Sex			1.000
Male	1555 (52.86)	7775 (52.86)	
Female	1387 (47.14)	6935 (47.14)	
Birth Year			1.000
2010	151 (5.13)	755 (5.13)	
2011	158 (5.37)	790 (5.37)	
2012	212 (7.21)	1060 (7.21)	
2013	251 (8.53)	1255 (8.53)	
2014	266 (9.04)	1330 (9.04)	
2015	361 (12.27)	1805 (12.27)	
2016	117 (3.98)	585 (3.98)	
2017	563 (19.14)	2815 (19.14)	
2018	464 (15.77)	2320 (15.77)	
2019	399 (13.56)	1995 (13.56)	
Birth Month			1.000
January	268 (9.11)	1340 (9.11)	
February	193 (6.56)	965 (6.56)	
March	235 (7.99)	1175 (7.99)	
April	247 (8.40)	1235 (8.40)	
May	216 (7.34)	1080 (7.34)	
June	247 (8.40)	1235 (8.40)	
July	214 (7.27)	1070 (7.27)	
August	266 (9.04)	1330 (9.04)	
September	286 (9.72)	1430 (9.72)	
October	279 (9.48)	1395 (9.48)	
November	241 (8.19)	1205 (8.19)	
December	250 (8.50)	1250 (8.50)	

Data are shown as number (%).

**Table 3:** Chi-square Analysis on Risk Factors of Neonates Acquiring Clinical Early-onset Sepsis.

Risk Factors	Sepsis Group (N = 2942)	Non-Sepsis Group (N = 14,710)	*p* Value
Maternal fever	40 (1.36)	51 (0.35)	<0.001
Chorioamnionitis	119 (4.04)	84 (0.57)	<0.001
Maternal genitourinary tract infections	205 (6.97)	349 (2.37)	<0.001
Maternal pneumonia	33 (1.12)	53 (0.36)	<0.001
Premature/prolonged rupture of membranes (PPROM)	1129 (38.38)	2965 (20.16)	<0.001
Delivery by Cesarean section	1928 (65.63)	8464 (57.54)	<0.001
Maternal systemic lupus erythematosus (SLE)	27 (0.92)	142 (0.97)	0.809
Maternal diabetes mellitus	214 (7.27)	879 (5.98)	0.008
Maternal antibiotics usage	1426 (48.47)	4655 (31.65)	<0.001
Steroid (rinderon or dexamethasone) usage	916 (31.14)	1853 (12.60)	<0.001
Birth body weight (g) *	1685 ± 612	2390 ± 539	<0.001
Gestational age (weeks)			<0.001
>32	1505 (51.15)	13,559 (92.17)	
28–32 (Very preterm)	999 (33.96)	903 (6.14)	
<28 (Extremely preterm)	438 (14.89)	248 (1.69)	
Mean ± SD	32 ± 3	35 ± 2	

Data are shown as number (% among total positive cases of this risk factor) unless stated otherwise. * presented as mean ± standard deviation.

**Table 4:** Conditional Logistic Regression on Risk Factors of Neonates Acquiring Clinical Early-onset Sepsis.

	Unadjusted Model			Adjusted Model (Full Model)			Adjusted Model (Stepwise Model)		
	OR	95% CI	*p* Value	OR	95% CI	*p* Value	OR	95% CI	*p* Value
Maternal fever	3.96	2.61 to 6.01	<0.001	4.46	2.57 to 7.73	<0.001	4.62	2.68 to 7.98	<0.001
Chorioamnionitis	7.34	5.54 to 9.73	<0.001	1.60	1.00 to 2.56	0.049	2.13	1.47 to 3.07	<0.001
Maternal genitourinary tract infections	3.08	2.58 to 3.68	<0.001	1.35	1.00 to 1.83	0.054			
Maternal pneumonia	3.14	2.03 to 4.85	<0.001	2.49	1.39 to 4.46	0.002	2.46	1.38 to 4.39	0.002
Premature/prolonged rupture of membranes (PPROM)	2.47	2.27 to 2.68	<0.001	1.85	1.66 to 2.06	<0.001	1.85	1.66 to 2.05	<0.001
Maternal antibiotics usage	2.03	1.88 to 2.20	<0.001	1.34	1.21 to 1.49	<0.001	1.35	1.21 to 1.50	<0.001
Steroid (rinderon or dexamethasone) usage	3.14	2.86 to 3.44	<0.001	1.26	1.11 to 1.42	<0.001	1.26	1.11 to 1.43	<0.001
Maternal diabetes mellitus	1.23	1.06 to 1.44	0.008	1.44	1.19 to 1.75	<0.001	1.44	1.19 to 1.75	<0.001
Maternal systemic lupus erythematosus (SLE)	0.95	0.63 to 1.44	0.812	0.68	0.42 to 1.10	0.118			
Delivery by Cesarean section	1.40	1.29 to 1.52	<0.001	1.17	1.05 to 1.30	0.004	1.17	1.05 to 1.30	0.004
Birth body weight (g) *	0.99	0.99 to 0.99	<0.001	0.99	0.99	<0.001	0.99	0.99 to 0.99	<0.001
Very preterm (Gestational age 28–32 weeks compared with >32 weeks)	9.97	8.98–11.07	<0.001	2.66	2.30–3.08	<0.001	2.67	2.31–3.09	<0.001
Extremely preterm (Gestational age < 28 weeks compared with >32 weeks)	15.91	13.50–18.76	<0.001	0.78	0.60–1.02	0.066	0.79	0.61–1.03	0.078

Abbreviations: OR, odds ratio; CI, confidence interval. * presented as mean ± standard deviation.

## Data Availability

The original contributions presented in this study are included in the article, and further inquiries can be directed to the corresponding author.
